# Recent Advances in Non-Destructive Testing Technology for Coated Steel Structure Welds

**DOI:** 10.3390/s25226923

**Published:** 2025-11-13

**Authors:** Zhiyong Ji, Dongsheng Xu, Honglun Wang, Junzhe Chen, Yunwei Fu

**Affiliations:** Xichang Satellite Launch Center, Xichang 615000, China; xds19880701@163.com (D.X.); whl_1689@163.com (H.W.); fywoec@163.com (Y.F.)

**Keywords:** coated steel structure, weld inspection, nondestructive testing, defect detection

## Abstract

The fabrication of a steel structure facility in the aerospace sector was executed through the implementation of welding techniques. In order to reduce the effects of environmental corrosion and extend its service life, it is typically coated with a protective layer. Nevertheless, conventional non-destructive testing (NDT) techniques generally necessitate preliminary procedures, such as coating removal and surface grinding, prior to inspection, leading to elevated costs and diminished efficiency. Consequently, the investigation into NDT methodologies for welds encased under coatings is of considerable practical significance. The objective of this paper is to comprehensively review and thoroughly analyze the latest research progress in NDT techniques for detecting defects in coated steel welds, seeking feasible approaches for achieving NDT on coated steel structures. Firstly, the paper examines the hazards of common weld defects and the challenges coatings pose to NDT operations. The text then proceeds to expound upon the principles, research advancements, and application scenarios of multiple NDT methods currently available for detecting defects beneath coatings. A comparative summary of these methods is provided, focusing on detection capabilities, coating penetration abilities, key advantages, and limitations. In conclusion, the paper provides insights into future development trends.

## 1. Introduction

Steel structures play a pivotal role in the development of modern infrastructure owing to their high strength, excellent toughness and ease of construction. They are used extensively in critical engineering projects such as bridges, sports stadiums, offshore platforms and aerospace projects. Welding, as the primary method for joining steel components, directly determines the overall safety and service life of the structure. However, steel structures usually have large components, complex designs, thick base materials and various weld configurations. Consequently, they cannot be fabricated, either fully or partially, using automated welding equipment, and manual multi-pass welding techniques are required instead. Various defects inevitably arise during the welding process, characterized by their high concealability, significant potential hazards, and considerable difficulty in rectification. Although welding quality inspections are conducted during production and prior to product delivery, it is inevitable that some defects may be missed or undetectable. Under load, these defects may propagate, ultimately leading to catastrophic accidents. Furthermore, operational loads and welding stresses may induce crack initiation within welds. Consequently, regular in-service inspection and maintenance of steel structure welds are paramount to ensuring the safe and stable operation of such facilities.

Steel structure facilities located in space launch sites usually face complex working conditions, such as environmental corrosion, high-temperature ablation and strong impact during operation. Therefore, in addition to conventional anti-corrosion coatings, thermal insulation coatings must also be applied to steel structure facilities to protect their service performance. According to the different working conditions, the thickness of the protective coatings is usually between 300 μm and 3 mm. While protective coatings are effective in protecting steel structures, they also present significant challenges in the detection of weld defects during service. NDT techniques, as vital tools for defect detection, have been widely applied in aerospace, petroleum, and marine industries, making weld defect inspection under coatings feasible [1]. However, in practical engineering applications, traditional NDT methods such as Ultrasonic Testing (UT) [2,3,4], Eddy Current Testing (ET) [5,6,7], Magnetic particle Testing (MT) [8,9,10,11] and Penetration Testing (PT) [10,12,13,14] usually require the coating to be removed during weld defect detection and have high requirements for surface roughness, which needs to be ground and polished. For instance, while traditional UT technology is widely adopted for its exceptional internal defect detection capability, existing application techniques lack coating penetration ability and require good surface coupling. Consequently, coatings on metal surfaces must be removed and surfaces ground smooth prior to inspection. Traditional ECT technology is primarily used for volumetric defect detection in facilities like oil pipelines. While this technique can detect defects at certain lift-off heights, the lift-off typically does not exceed 0.5 mm, imposing high flatness requirements on the inspected surface. Magnetic particle testing (MT) and penetrant testing (PT) rely on visual inspection to identify surface defects. Consequently, coating removal and surface finishing are required before testing to enable defect detection using magnetic suspension liquid (MT) or fluorescent dye (PT). The aforementioned existing NDT methods not only increase inspection costs and reduce efficiency but also introduce new risks and safety hazards due to secondary coating repairs. Conversely, failing to remove coatings prior to inspection leads to severe signal attenuation, increased interference, reduced detection sensitivity, and even the formation of detection blind spots, making accurate assessment of weld defects beneath coatings exceptionally challenging.

Effectively inspecting welded joints in steel structures while preserving coatings is pivotal to achieving rapid and accurate defect localization. This allows defective areas to be detected and rectified in a timely manner, significantly mitigating the risk of safety incidents. These capabilities offer substantial economic and engineering benefits, including safeguarding facility integrity, extending service life and reducing maintenance costs. However, research and application of non-destructive testing methods for coated steel welds remain limited, with no existing review articles on this topic. Therefore, this paper summarizes the research progress and applications of NDT technologies capable of penetrating coatings. It aims to explore methods for effectively inspecting steel structural welds without removing coatings, providing a reference for scholars or technicians facing similar challenges.

In this paper, the related technologies for NDT of welds in coated steel structures will be systematically explored. Section 2 first examines the causes and preventive measures for common weld defects, analyzing the challenges coatings pose to NDT operations and proposing countermeasures. Section 3 then details the principles, research progress, and application status of multiple NDT methods currently available for detecting weld defects beneath coatings. Section 4 summarizes and compares different methods based on detection capability, coating penetration ability, key advantages, and limitations, identifying suitable NDT techniques for weld defect detection in coated steel structures within a specific aerospace field. Section 5 outlines future development trends. Finally, Section 6 provides a comprehensive summary of the entire paper.

## 2. Analysis of Weld Defect Detection Under Coating

### 2.1. Typical Weld Defects

Welding is a sophisticated thermal processing procedure entailing the melting, metallurgical reactions, and solidification of metals. It is imperative to acknowledge that, throughout the course of the welding process, the occurrence of various discontinuities and defects within the weld and its surrounding regions is a possibility. The presence of these defects can be attributed to a multitude of factors, including but not limited to: the inappropriate selection of welding process parameters; inadequate operational skills on the part of the welding operator; quality issues with welding materials; or inherent defects in the base metal itself. These defects have been shown to diminish the mechanical properties of the welded joint, thereby affecting the structure’s load-bearing capacity and service life. The categorization of defects is typically based on their location within the weld, with surface defects and internal defects being the most common categories.

**Figure 1:**
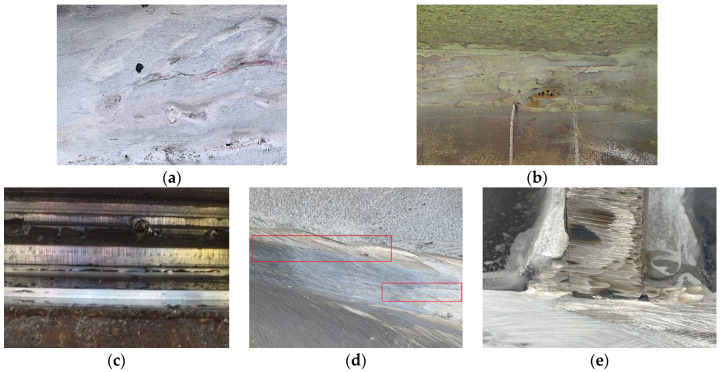
Common weld defects. (**a**) Crack. (**b**) Porosity. (**c**) Slag inclusion. (**d**) Incomplete fusion, The red box indicates the defect location. (**e**) Incomplete penetration.

Surface defects manifest on the weld surface or base metal surface and are generally observable to the naked eye or with low-power magnification. Common surface defects include surface porosity, surface cracks, crater defects, and burn-through. The presence of internal defects within the weld structure is not discernible through visual inspection; consequently, the utilization of non-destructive testing or destructive examination is imperative for their identification. Common internal defects include incomplete fusion, internal porosity, internal cracks, slag inclusions, and incomplete penetration [15,16]. Among these defects, area-type defects, such as cracks, incomplete fusion and penetration, alongside volume-type defects, such as porosity and slag inclusions, pose significant hazards. It is imperative to direct particular attention to these defects during the production and service life of steel structures [17]. The various forms of these defects are illustrated in Figure 1. Consequently, the present section is devoted to an examination of the characteristics, formation mechanisms, and hazards of such defects.

Firstly, the defects generated during the manufacturing process will be analyzed.

#### 2.1.1. Cracks

Cracks generated during welding are fissures that form within welds or the heat-affected zone of the base metal. They are caused by the disruption of metallic atomic bonding forces. These fissures are categorized into two distinct types: hot cracks and cold cracks [18,19]. The formation of hot cracks is predominantly observed during the welding process or when the weld cools to elevated temperatures, with a tendency to occur along grain boundaries. The formation of these compounds is chiefly associated with low-melting-point eutectic compounds formed by impurities such as sulfur and phosphorus in steel. Such compounds are also formed during the process of welding, and as a result of crystallization segregation. Their prevalence is commonly observed in the welding of austenitic stainless steels, high-carbon steels, and certain alloy steels. The phenomenon of cold cracks is characterized by the formation of fissures in the weld as it undergoes a reduction in temperature. These fissures may also develop subsequent to the welding process, exhibiting a delayed onset. The formation of these structures is primarily associated with the combined effects of hardened microstructure, hydrogen content, and restraint stresses [20,21]. Cracks represent the most hazardous type of welding defect, exhibiting extremely high stress concentration at their tips. These materials have been observed to exhibit a propensity for rapid propagation under both dynamic and static loading conditions. This tendency, when coupled with the material’s inherent fragility, can result in the sudden and unexpected failure of the structure, potentially leading to significant accidents.

#### 2.1.2. Porosity

Porosity emerges when gases entrapped within the weld pool do not completely escape prior to metal solidification, resulting in the formation of cavities within the weld metal. These porosities may be classified as either surface or internal porosity, manifesting as isolated or densely clustered defects. The primary causes of welding defects include the following: moisture contamination of welding consumables; contamination of the base metal surface with oil, rust, or moisture; insufficient purity or improper flow rate of shielding gas; excessive welding speed; or an overly long arc. These factors cause gases to dissolve into the molten pool metal. Upon reaching a state of solidification, the reduced solubility of these gases leads to their precipitation, resulting in the formation of bubbles [22,23,24,25]. The presence of porosity in the weld reduces the effective load-bearing cross-sectional area, thereby diminishing its strength and ductility. This is particularly evident in cases of dense or chain-like porosity, where joint performance is significantly compromised.

#### 2.1.3. Slag Inclusion

Slag inclusions are non-metallic impurities that are retained within weld metal, forming point-like, linear, or block-shaped defects. These phenomena primarily manifest during multi-layer, multi-pass welding processes, such as stick electrode arc welding and submerged arc welding [26,27]. The underlying causes of this phenomenon have been identified as follows: inadequate removal of interpass slag; insufficient welding current, resulting in high slag viscosity and poor flotation; narrow fillet angles or excessively narrow weld beads, trapping slag; and improper electrode manipulation. The presence of slag inclusions has been shown to disrupt the continuity of the metal, leading to stress concentration and a reduction in the weld’s strength, ductility, and toughness. Of particular concern are sharp-edged inclusions, which pose a significant hazard [28,29].

#### 2.1.4. Incomplete Penetration

Welding incomplete penetration is characterized by the failure of the base metal to melt during the welding process, thereby impeding the penetration of the weld metal into the root of the joint. Alternatively, incomplete penetration between layers during multi-pass welding can result in the formation of defects [30,31]. The primary causes of this phenomenon include insufficient welding current, resulting in shallow penetration; excessive welding speed; inadequate bevel angle, insufficient root gap, or excessive blunted edge; excessive electrode diameter; and improper operation. The absence of fusion defects has been demonstrated to result in a substantial reduction in the effective load-bearing area of the welded joint, consequently leading to a notable decrease in joint strength. Sharp notches are observed to form at the unfused root region, thereby creating areas that are highly susceptible to stress concentration and crack initiation [14,32,33].

#### 2.1.5. Incomplete Fusion

Incomplete fusion defects, akin to incomplete penetration, emerge from incomplete fusion between the weld bead and base metal or between weld beads themselves. The primary causes of this phenomenon include: excessively low welding current or excessive welding speed; inadequate removal of scale, oil, or other contaminants from the groove or weld surface; improper electrode angle, preventing arc focus on the fusion zone; and magnetic blow effects. The presence of such defects has been shown to have a substantial impact on the load-bearing capacity and fatigue strength of the joint, readily becoming a crack initiation point [34,35,36].

#### 2.1.6. Preventive Measures

The five types of defects caused by production can be addressed by enhancing welding quality inspection and repair during the manufacturing process. Defects arising during the service life of facilities and equipment predominantly manifest as cracks. These defects originate from two primary causes: First, defects or stress concentrations introduced during production create weak points in welds, which then develop cracks under load during service. Preventing this type of crack involves measures similar to those for the five manufacturing-related defects mentioned above. Additionally, stress concentration points must be inspected during manufacturing, and post-production heat treatment or equivalent methods should be applied to eliminate stresses concentrated during welding. The other type arises from fatigue cracks in welds caused by alternating loads during service. For these cracks, equipment health monitoring or regular non-destructive testing is essential to detect and promptly repair defects. This ensures the safe and stable operation of facilities and equipment while extending their service life.

### 2.2. Challenges Presented by Coatings to Non-Destructive Testing

Performing non-destructive testing on steel structure welds whilst retaining the coating offers significant advantages. On the one hand, it substantially enhances testing efficiency and shortens inspection timelines, facilitating the timely detection of defects in equipment and facilities. This enables effective maintenance of infrastructure and prevents the occurrence of safety incidents. On the other hand, preserving the coating avoids the risks and potential hazards associated with reapplying protective coatings, whilst also reducing expenditure. This approach thus delivers considerable benefits in terms of both safety and economy. However, retaining the coating also presents substantial challenges for the inspection process, primarily manifested in the following aspects: Preserving coatings during inspection poses considerable challenges, chiefly manifested in the following aspects:

1. The problem of signal attenuation and distortion becomes more pronounced. Most NDT methods rely on some form of energy wave interacting with the material being tested, such as electromagnetic waves or ultrasonic waves. Coating materials typically exhibit distinct physical properties from steel, including conductivity, permeability, acoustic impedance, and dielectric constant. This leads to significant absorption, scattering, and reflection of energy waves as they traverse the coating. Consequently, signal strength is substantially attenuated, signal-to-noise ratio is reduced, and signal distortion may occur. These factors render the faint signals originating from weld defects difficult to effectively receive and identify.
2. The lift-off effect becomes more obvious. For techniques relying on close coupling between the probe and the surface under test, the presence of a coating effectively increases the distance between the probe and the steel substrate, i.e., the lift-off distance. This drastically reduces detection sensitivity, with particularly severe impacts on high-frequency signals.
3. Interface reflections and false defect signals become more prominent. The interface formed between the coating and the steel substrate may generate intense reflection signals or mode conversion, which may be misinterpreted as defect signals or mask genuine defect signals, thereby increasing the difficulty of interpretation.
4. Coupling becomes more challenging. For techniques such as ultrasonic testing that require effective acoustic coupling, surface irregularities or unevenness in the coating, or poor compatibility between the coating material itself and the coupling agent, can lead to difficult or unstable acoustic coupling. This adversely affects the reliability and repeatability of the test results.

Due to the aforementioned factors, the precise localization and quantitative assessment of defects in welded joints of coated steel structures using conventional NDT techniques prove exceptionally challenging. Indeed, the vast majority of traditional inspection methods prove incapable of effectively detecting such defects when confronted with these circumstances. To address these challenges, the following approaches can be considered:

1. To mitigate reduced detection accuracy caused by signal attenuation and distortion, signal strength can be enhanced through parameter optimization, improved sensor design, and selection of appropriate detection methods, thereby improving detection capability [37,38,39,40,41,42,43].
2. For decreased detection sensitivity or pattern conversion caused by lift-off effects, interface reflections, and false defects, detection accuracy can be improved by optimizing probe design, employing lift-off compensation techniques, applying signal processing algorithms, and utilizing feature extraction and pattern recognition technologies [44,45,46,47,48,49].
3. For problems requiring effective interface coupling, advanced non-contact NDT techniques must be pursued through specialized probe design and technological innovation to achieve detection of metal substrate defects beneath coatings [50,51,52,53].

Based on the above analysis, to identify reliable technologies suitable for detecting weld defects in coated steel structures, Section 3 reviews the key characteristics and research progress of existing NDT techniques.

## 3. Weld Defect Detection Technology Beneath Coatings

Regarding NDT of coated steel structures, scholars and engineers have conducted a series of studies. For instance, technologies such as Pulse Eddy Current Testing (PEC), Microwave Testing (MWT), Electromagnetic Acoustic Transducer (EMAT), and thermal imaging testing have demonstrated significant potential in penetrating coatings of certain thicknesses. The following primarily reviews current non-destructive testing techniques capable of detecting defects beneath coatings, focusing on methods based on electromagnetic, acoustic, microwave, and thermal imaging technologies.

### 3.1. Eddy Current Testing (ECT)

#### 3.1.1. Inspection Principle

**Figure 2:**
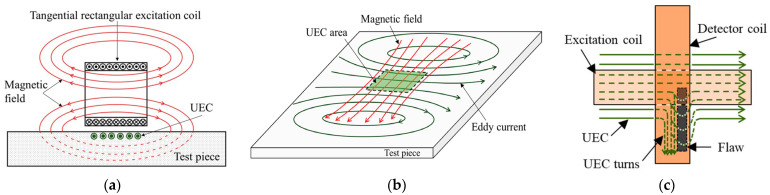
Principle of ECT [58]. (**a**) Electromagnetic induction generates eddy currents. (**b**) Magnetic field and eddy currents on the specimen surface. (**c**) Inspection of surface defects on test specimens.

Eddy Current Testing operates on the principle of electromagnetic induction. When a coil carrying an alternating current is brought near conductive material, vortex-like currents—known as eddy currents—are induced within the material. The distribution and magnitude of these eddy currents are influenced by the material’s electrical conductivity, magnetic permeability, geometric shape, and the presence of defects [54,55,56,57]. By measuring changes in the coil’s impedance or induced voltage caused by these eddy current variations, defects can be detected, as illustrated in Figure 2. ECT offers the advantages of being non-contact and rapid, making it highly suitable for on-site screening.

Moreover, a significant advantage of ECT is its ability to detect defects in the metallic substrate through non-conductive coatings without requiring removal of surface paint or anti-corrosion layers. This is because coatings are typically non-conductive materials that offer minimal impedance to high-frequency electromagnetic fields, allowing eddy currents to be induced within the metallic workpiece. Consequently, in steel structure weld inspection, eddy current probes can directly scan the coated surface to detect cracks and other defects within the weld zone. 

#### 3.1.2. Recent Advances

Regarding the detection of defects in the metal substrate beneath coatings, AbdAlla et al. [37] summarized the structural design factors of eddy current probes affecting crack detection accuracy, alongside past efforts by researchers to mitigate the impact of lift-off effects on crack detection. Ali et al. [59] investigated the impact of metallic paint layer thickness (650–1210 μm) on detecting weld cracks in stainless steel and carbon steel using ECT. Experiments were conducted on carbon steel and stainless steel plates, with artificial cracks measuring 1 mm in width,3 mm in depth, and 39 mm in length positioned at identical locations—the weld toe, root, and heat-affected zone—of welds on both plate types. Results indicated that at an operating frequency of 100 Hz, the detectable length of identical crack defects progressively decreased with increasing metallic paint layer thickness. To mitigate the lifting effect from coatings, Ali [44] proposed an Error Compensation ECT (ECECT) system featuring a high sensitivity and signal-to-noise ratio. To achieve lifting compensation, this system simultaneously employs both absolute probes (AP) and differential probes (DP). Furthermore, Mamdani fuzzy logic is applied to process the phase, amplitude, and loop width of the detection signal, thereby determining the crack dimensions. Experiments compared the proposed method with conventional detection techniques, demonstrating over double the accuracy in crack depth measurement. Additionally, the method validated measurement errors for 1–5 mm deep cracks under 1–4 mm coating thickness at 3 kHz, 6 kHz, and 9 kHz frequencies, revealing the lowest measurement error at 3 kHz. Cheng et al. [38] designed a novel eddy current sensor for detecting surface cracks beneath metallic coatings. Based on mutual induction principles, the sensor features a single-turn Koch curve excitation coil and a multi-turn Koch curve signal pickup coil. Both excitation and signal pickup coils have an outer diameter of 10 mm, with excitation coil wire width of 0.2 mm and signal pickup coil wire width of 0.05 mm. Simulation studies investigated eddy current distribution evolution with depth, revealing decreasing eddy current density and reduced concentration with increasing depth. Actual defect detection results demonstrate that at a 10 kHz operating frequency, this sensor can detect surface cracks (0.25 mm wide, 3 mm deep) in the base material beneath 2 mm-thick aluminum plates. Furthermore, this sensor can largely disregard the influence of crack orientation on detection outcomes. Alexey et al. [60] applied ECT to crack detection in large-diameter pipes with maximum coating thicknesses of 10 mm. In this study, a 32-channel eddy current tester enabled automated inspection of large-diameter pipes with wall thicknesses of 39 mm and diameters of Ø813 mm. Experiments demonstrated that the inspection equipment, operating at a frequency of 70 kHz, could not only successfully detect localized areas with 20% differences in base material hardness but also achieve crack detection.

Overall, ECT stands as one of the effective methods for detecting surface cracks in metal substrates beneath coatings. Its ability to penetrate coating thicknesses depends on the dielectric properties of the coating and the excitation frequency, generally enabling reliable detection of surface defects beneath non-conductive coatings several millimeters thick. Additionally, the penetration depth of eddy current testing is inversely proportional to the square root of frequency. Traditional high-frequency methods are largely ineffective for detecting defects in steel substrates beneath coatings, typically requiring frequencies below 10 kHz. However, sensitivity decreases correspondingly as frequency is reduced. Some advanced ECT techniques achieve coating-penetrating defect detection in coated metal structures at frequencies between 10 kHz and 100 kHz, balancing coating penetration capability with detection sensitivity. Low-frequency ECT exhibits sensitivity to surface-opening cracks and near-surface defects, though its quantitative accuracy is significantly influenced by lift-off effects and signal processing algorithms. To better distinguish coating thickness variations from defect signals, research into multi-frequency eddy current techniques, novel eddy current probes, or advanced signal processing technologies may be considered.

### 3.2. Pulsed Eddy Current (PEC)

#### 3.2.1. Inspection Principle

Pulse Eddy Current testing represents an advanced technique within eddy current inspection. Unlike conventional single-frequency or multi-frequency eddy current methods, PEC employs square-wave or step-pulse excitation to generate a broad-spectrum electromagnetic field. Its detection signal encompasses a rich array of frequency components—including both high-frequency elements for surface defect detection and low-frequency elements capable of identifying deeper defects. This overcomes the limitations of conventional eddy current testing, offering the advantage of obtaining multi-level defect information in a single inspection. Furthermore, its electromagnetic non-contact coupling characteristic enables rapid, large-area inspection capabilities [61,62,63]. Moreover, PEC technology effectively suppresses the lift-off effect, enabling penetration through non-conductive coatings, such as anti-corrosion layers or thermal insulation. It is primarily employed to detect corrosion thinning and near-surface defects beneath coatings, though its capability to detect deeply buried defects within welds remains limited [64,65]. Its coating penetration capacity is primarily influenced by coating material properties, substrate electrical and magnetic conductivity, and excitation pulse parameters. Moreover, PEC imposes no stringent requirements on the surface being tested, necessitating only a relatively clean surface.

#### 3.2.2. Recent Advances

In recent years, pulsed eddy current technology has advanced rapidly. Researchers have enhanced the signal-to-noise ratio by optimizing pulse waveforms and employing high-sensitivity sensors such as Hall elements or coil arrays. Nafiah et al. [45] employed principal component analysis (PCA) to process pulsed eddy current detection signals, thereby mitigating the impact of composite cladding layer thickness using conductive and insulating materials on detection results. Experiments employed non-conductive materials 40 mm thick and conductive metals ranging from 0.5 to 1.5 mm (in 0.5 mm increments) as composite coatings. The results confirmed that PCA effectively suppressed coating-induced signal interference during steel plate thickness reduction detection. Jiang et al. [39] designed a flat U-shaped sensor based on PEC technology for detecting defects in steel pipes with protective steel sleeves. The protective layer consisted of 7.5 mm thick carbon steel, positioned 34.5 mm away from the pipe. Comparing results with a traditional coaxial cylindrical probe revealed that the U-shaped probe effectively suppressed noise. Parameters for both probes are shown in Table 1. Song et al. [66] proposed the Time to Last Peak Point (TLPP) based on predictive signal analysis as an effective characteristic for measuring defects in non-ferromagnetic metal components under high lift-off conditions. Multiple experimental series validated the impact of varying coating thicknesses on PEC defect detection outcomes. The coatings comprised 0.5 mm-thick metal sheets over insulating materials between 6 and 14 mm thick. Results confirmed this methodology suppresses coating effects, limiting detection errors to within 11%.

Further studies have combined pulsed eddy current with numerical simulation to quantitatively assess defect depth and dimensions. Xu [46] derived the eddy current skin depth formula and established an analytical model for T-R type eddy current probes to analyze the impact of varying lift-off distances and specimen thicknesses on detection capability. In this study, numerical simulation optimized the excitation and detection coil parameters of the T-R probe. Two probe designs with different dimensions (shown in Table 2) were developed, and their defect detection capabilities at various lift-off distances were experimentally validated. The results indicate that Probe P1 can detect Ø4 mm circular holes with depths ranging from 1 to 8 mm at a 1 mm lift-off distance. At a 20 mm lift-off distance, it effectively detects square holes with depths from 1 to 8 mm and side lengths of 20 mm, though its dimensional resolution in the depth direction is limited. The P2 probe performed better in the same experiments. Yang et al. [40] proposed a U-shaped magnetic conductor focusing probe based on pulsed eddy current technology to address the detection blind zone issue of cylindrical probes. The excitation section of this probe is wound on one leg of a U-shaped ferrite core with electrical conductivity of 240 S/m and relative magnetic permeability of 5628. The wire diameter is 0.57 mm, with 238 turns, an inductance of 3.44 mH, and a resistance of 0.995 Ω. Two detection coils are connected in a differential configuration. Each detection coil has a wire diameter of 0.1 mm, 3400 turns, and a resistance of 184.39 Ω, with an inductance of 32 mH. Numerical simulation was employed to study the magnetic field distribution around the probe excitation coil, analyzing how magnetic conductors guide the magnetic field. Simultaneously, the focusing of induced eddy currents on the test specimen at different probe lift-off heights was compared to evaluate the probe’s sensitivity for detecting localized defects. Experiments demonstrated that the probe accurately detected wall thickness reductions down to 0.5 mm at a lift-off height of 10 mm, while maintaining detection of 30 mm in width and length, 1.5 mm in depth reductions at 50 mm lift-off, indicating high detection precision.

The aforementioned research findings demonstrate that pulsed eddy current testing finds extensive application within the petrochemical industry, such as for detecting corrosion defects in pipelines and storage tank walls clad with insulation or anti-corrosion coatings. Regarding weld inspection, pulsed eddy currents can be employed to detect internal cracks near the heat-affected zone or buried defects at the weld root, proving particularly suitable where coating removal is impractical or inspection space is constrained. It is worth noting that pulsed eddy current exhibits slightly lower sensitivity for detecting planar defects (such as cracks) compared to volumetric defects (such as slag inclusions or porosity). Consequently, it is often employed in conjunction with other methods for crack detection in welds.

### 3.3. Magnetic Flux Leakage (MFL)

#### 3.3.1. Inspection Principle

**Figure 3:**
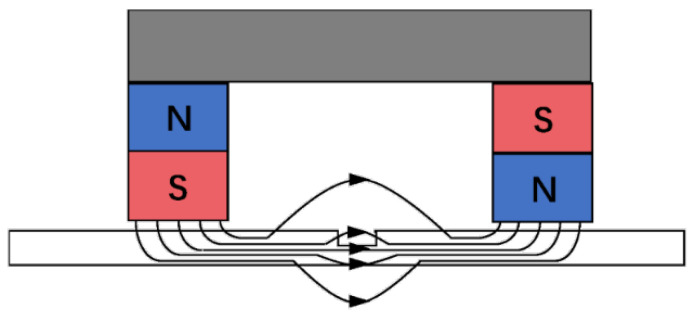
Principle of MFL [67].

Magnetic Flux Leakage detection is an electromagnetic inspection method evolved from magnetic particle testing, serving as a widely employed non-destructive testing technique for ferromagnetic materials [67]. Its principle involves magnetizing a portion of the workpiece to saturation or near-saturation using permanent magnets or electromagnets. Should surface or near-surface defects (such as cracks or inclusions) exist, increased magnetic resistance at these flaws causes magnetic flux lines to escape from the workpiece surface, generating a leakage magnetic field. By placing magnetic sensors (such as Hall elements or coils) on the workpiece surface to detect variations in these leakage magnetic fields, the location and dimensions of defects can be determined [68]. The operational principle is illustrated in Figure 3. Leakage magnetic detection typically requires sensors to be positioned close to the workpiece surface to capture the leakage field. However, for thin non-magnetic coatings (such as paint), the leakage field can still penetrate the coating and be detected by the sensor. Therefore, in practical applications, workpieces with thin coatings can undergo leakage magnetic field detection directly without coating removal.

#### 3.3.2. Recent Advances

Traditional MFL has primarily been employed for identifying corrosion-related defects, though in recent years, it has also been applied to research concerning weld seam defects. Hayashi et al. [69] designed an Unsaturated Alternating Current MFL (USAC-MFL) probe to address the common issue where conventional MFL probes are susceptible to the influence of welded structure geometry and non-uniform magnetic permeability during weld inspection. This probe consists of two ferrite cores: an induction coil (25 turns) and a tunnel magnetoresistance (TMR) sensor. The TMR sensor is mounted on a flexible circuit board, enabling angle adjustment to detect different weld regions: the weld itself or the weld toe. This probe exhibits low power consumption and high sensitivity when detecting cracks in welded areas, distinctly differing from conventional MFL systems that require high currents and thus high energy consumption for crack detection. Experiments were conducted on 20 right-angle weld specimens with surface coating thicknesses of 125 μm and 250 μm. Forty-eight cracks, each 1–5 mm deep, were introduced into the weld and weld toe regions. Results demonstrated that the probe achieved detection rates and accuracy exceeding 80% for surface and near-surface cracks beneath 250 μm coatings. Wu et al. [47] developed and tested a ferrite-core-based MFL sensor that effectively suppresses lift-off, applying it to MFL inspection of wellhead drill pipes. This sensor was applied to MFL inspection of wellhead drill pipes. It consists of an induction coil and a ferrite core wound with an insertion coil made of enameled wire. Compared to Hall elements, the induction coil avoids saturation issues under strong magnetic fields. Experiments were conducted on a hollow drill pipe with a diameter of 127 mm, which was machined with two sizes of circumferential cracks and two sizes of hole-like defects. One crack type measured 1 mm in width and depth, with a length of 25 mm, while the other measured 1 mm in width, 0.5 mm in depth, and 25 mm in length. The hole-like defects had diameters of 1.6 mm and 3.2 mm, respectively. The results demonstrated that, at a detection speed of 20 m/min, the sensor accurately detected both cracks and hole defects at a lift-off distance of 5 mm.

Numerous scholars have also made significant contributions towards suppressing lift-off interference. Jia et al. [3] proposed a novel filtering method to mitigate the impact of lift-off effects in MFL detection of rail defects. This approach employs an array sensor configuration, leveraging distinct signal characteristics in the x and z directions to effectively suppress interference signals induced by lift-off forces. Experiments demonstrate that this method reliably maintains detection accuracy for surface defects when the lift-off height ranges between 2 mm and 3.5 mm. Peng et al. [41] analyzed the approximate negative exponential relationship between defect magnetic flux leakage signals and lift-off using the magnetic dipole analysis method, establishing a simplified numerical calculation model for flux leakage signals based on lift-off. They also proposed a lift-off correction method incorporating an exponential compensation mechanism that focuses solely on the lift-off correction value. Tests were conducted on a Ø27.3 mm steel pipe with a wall thickness of 12.7 mm, using defect sizes as shown in Table 3. Results indicate this method effectively mitigates signal interference caused by a 2 mm lift-off height. Wang et al. [70] proposed an analytical method to linearize the lift-off effect, deriving spatial analytical expressions through magnetic dipole theory and Fourier transform techniques. Experiments were conducted on a metal plate measuring 160 mm in length, 100 mm in width, and 7 mm in thickness. Artificial defects were introduced into this metal plate (dimensions as shown in Table 4). The lift-off range spanned 0.5–8 mm, validating the impact of lift-off height on detection results. The results demonstrate that the lift-off effect in MFL exhibits excellent linear quantization characteristics in the spatial domain. To achieve defect detection in subsea pipelines, Qu et al. [71] proposed a defect estimation method based on axial peak-to-valley values and radial peak-to-peak distances. This approach leverages the axial symmetry of the axial component and the central symmetry of the radial component of the MFL magnetic field to achieve defect size inversion. Experiments demonstrate that for detecting rectangular defects in pipelines, a lift-off height between 3 and 5 mm represents the optimal range balancing vibration interference and detection accuracy. In practical applications, non-conductive coatings on ferromagnetic substrates can be approximated as lift-off heights, making these studies potentially applicable to weld inspection beneath coatings in steel structures.

Past research clearly demonstrates that MFL detection enjoys extensive industrial application, particularly in the inspection of oil and gas pipelines and storage tanks. Pipeline internal inspection pigging systems widely employ flux leakage technology, enabling the detection of corrosion and crack defects on both inner and outer pipe wall surfaces during operation, whilst pinpointing their precise locations. For coated welds, MFL detection can identify surface and near-surface defects such as cracks and porosity, though it exhibits limited sensitivity towards deeper-seated cracks. MFL testing equipment is simple, is reliable, and offers rapid inspection speeds, making it suitable for large-area screening. However, the process typically requires magnetizing the workpiece, which may lead to uneven magnetization issues on complex-shaped welds. Overall, MFL remains one of the effective methods for detecting surface defects in ferromagnetic materials beneath coatings, enabling the swift identification of defects compromising structural integrity while ensuring coating integrity.

### 3.4. Alternating Current Field Measurement (ACFM)

#### 3.4.1. Inspection Principle

**Figure 4:**
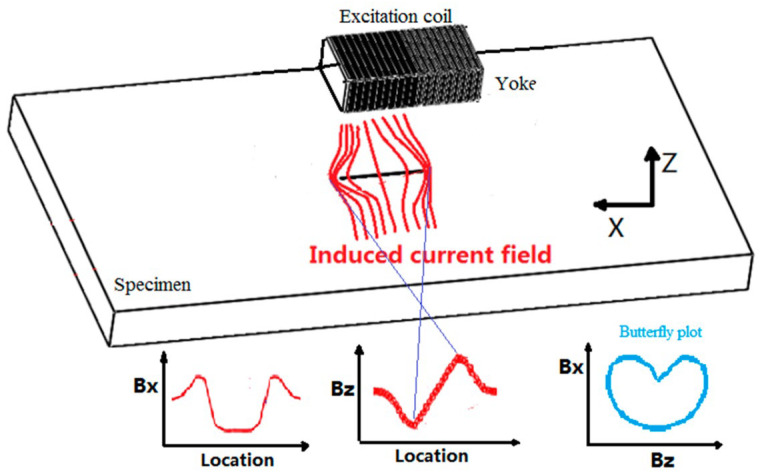
Principle of ACFM [77].

ACFM technology is a non-contact detection technique based on electromagnetic induction, representing a variant of MFL. It can identify metallic defects and determine their length and depth dimensions [72,73]. This technology operates by applying an alternating current signal to an excitation coil. When the probe scans near the specimen surface, a uniform alternating current field is generated at the surface. When this current field encounters a defect, distortion occurs, leading to increased current density at the defect’s edges and reduced current density at its base. Consequently, a leakage magnetic field forms around the defect. By measuring variations in this leakage field using a probe positioned above the workpiece, the location and dimensions of cracks can be determined [74,75,76]. Its operating principle is illustrated in Figure 4.

A notable advantage of ACFM technology is its ability to detect defects in the metal substrate beneath coatings without requiring pre-treatment such as coating removal or surface cleaning. It is primarily employed for detecting surface cracks and lack of fusion defects in welds. The inspection probe typically maintains a distance of several millimeters from the workpiece surface; provided the coating thickness remains within permissible limits (generally up to several millimeters), it does not significantly impair detection performance. However, if coating irregularities on the inspected surface cause substantial variations in lift-off height, this may still affect detection outcomes.

#### 3.4.2. Recent Advances

In recent years, experts and scholars have conducted extensive research to mitigate lift-off effects influenced by coatings and other factors. Yuan et al. [48] investigated signal distortion mechanisms under random and constant lift-off variations during ACFM testing, providing theoretical guidance for crack detection and evaluation under lift-off conditions. Subsequently, a novel array image feature method was proposed. This technique distinguishes crack characteristic signals from delamination interference signals based on temporal and spatial variations in response signals acquired by a novel longitudinal array ACFM probe. Using test specimens machined with ten cracks (two sets of 2 mm-wide cracks with varying lengths and depths; specific dimensions shown in Table 5), experiments verified the impact of random lift-off heights ranging from 1 to 5 mm on detection results. Results indicate that under random lift-off heights, the maximum relative errors for crack length and depth detected by this method are 8.56% and 8.63%, respectively, demonstrating its effectiveness. To address the impact of surface contaminants on ACFM crack detection in metals, Zhao et al. [78] comprehensively analyzed the relationship between lift-off height and Bx signal variation using finite element methods and experiments. In experiments, A4 paper simulated non-conductive coatings: uniform 1–4 mm thicknesses were tested, while random 1–4 mm lift-offs simulated non-uniform thicknesses. Results indicate optimal detection performance at an excitation frequency of 800 Hz, where relative evaluation errors for crack depth and length fluctuate within ±10%, with a uniform crack width of 0.5 mm. Other dimensions are shown in Table 6. To address the impact of coating corrosion and surface irregularities on crack detection accuracy due to varying lift-off heights, Zhao et al. [79] proposed an advanced lift-off effect compensation algorithm based on ACFM technology. This compensation algorithm comprises three key steps—R solution approach, lo inference, and establishing a magnetic characteristic surface based on z-axis magnetic signals—enabling crack size measurement at arbitrary lift-off distances. In this study, non-conductive coatings were simulated by applying rubber layers over aluminum substrates with 0.8 mm wide cracks. Three thicknesses (1.5 mm, 3.5 mm, and 5.5 mm) were simulated while maintaining a stable 4 mm lift-off distance for the scanning probe. This setup validated the lift-off compensation algorithm, with crack dimensions shown in Table 7. The results indicate that the algorithm can control the maximum relative error in defect depth within 10%.

Additionally, some scholars have made contributions to the practical application of ACFM technology. Yuan et al. [80] proposed a Dual Gradient Fusion Algorithm (DGFA) based on ACFM to detect irregular cracks in steel structures without coating removal. This algorithm generates a fusion gradient field image by processing the background signal of the Bz gradient field and normalizing the gradient field. In this study, the distorted electric field around irregular cracks was first analyzed using the finite element method, followed by experimental validation of the algorithm. The results demonstrate that DGFA technology can effectively detect a crack measuring 0.8 mm in width, 2 mm in depth, and 20 mm in length beneath a 2 mm polyethylene coating, while ignoring the influence of crack orientation. This finding aligns with simulation results. Guo et al. [81] addressed reduced detection accuracy in subsea pipeline crack inspection using ACFM, caused by variations in lift-off distance due to coating thickness changes and surface irregularities, by proposing an algorithm to suppress lift-off effects. This algorithm determines a new excitation signal based on the phase angle of the lift-off (LOI) at the time-domain intersection point of the original detection signal, and was validated through simulation and experimentation. The results demonstrate that within a peel distance range of 1 to 4 millimeters, this method effectively suppresses the influence of peel effects, significantly reducing interference from variations in coating thickness on crack size and depth measurements for cracks with a width of 0.5 mm, depth of 3 mm, and length of 20 mm.

Overall, ACFM represents an efficient and reliable method for detecting surface cracks in metallic substrates beneath coatings, enabling inspection without halting production or damaging the coating while ensuring structural integrity. It should be noted that ACFM is primarily suited for detecting surface cracks in metallic materials, with limited capability for internal defects. Furthermore, the probe must be maintained at an appropriate distance and angle from the weld, necessitating a certain level of operator skill. Future developments may explore integrating ACFM with other electromagnetic techniques, such as pulsed ACFM, which holds promise for enhancing detection capabilities regarding subsurface defects. Furthermore, automated signal recognition and imaging for ACFM represent another avenue of research, potentially reducing reliance on operator expertise.

### 3.5. Ultrasonic Testing (UT)

#### 3.5.1. Inspection Principle

**Figure 5:**
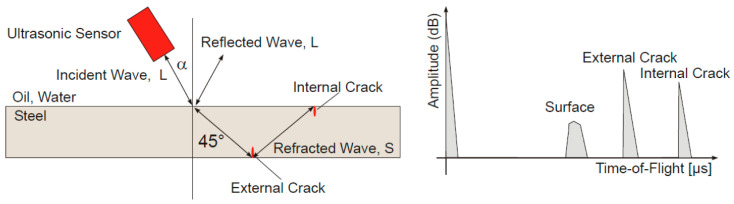
Principle of UT [15].

Ultrasonic Testing is a non-destructive testing method that utilizes the propagation characteristics of ultrasonic waves within materials to detect internal defects. The ultrasonic probe converts electrical pulses into ultrasonic pulses which are transmitted into the material under examination. These ultrasonic waves propagate through the material at a specific velocity. Upon encountering an interface with altered acoustic impedance, reflection occurs. The reflected waves are received by the same or another transducer and converted back into electrical signals [82]. By analyzing the time, amplitude, and waveform of the echoes, the location, size, and nature of defects can be determined. The underlying principle is illustrated in Figure 5. UT can detect various internal defects in workpieces, such as cracks, lack of fusion, and porosity, exhibiting particular sensitivity to surface-type defects like cracks, lack of fusion, and lack of penetration. This technique offers advantages including excellent localization and quantification capabilities, along with portable equipment. However, it also faces numerous challenges when inspecting welded joints in steel structures with protective coatings [83,84,85]:

- Coating attenuation and interface issues: This represents the foremost challenge in detecting weld defects beneath coating layers. The significant disparity in acoustic impedance between non-conductive coatings and steel, coupled with their high attenuation coefficients, causes severe energy loss and scattering of ultrasonic waves. This results in a drastic reduction in signal-to-noise ratio, thereby obscuring genuine signals from weld defects or leading to their misinterpretation as defects.
- Coupling issues: Conventional UT necessitates the use of coupling agents to eliminate air gaps between the probe and the surface being inspected, ensuring effective transmission of ultrasonic energy into the workpiece. When the surface roughness of protective coatings is suboptimal, this leads to difficult and unstable coupling, compromising the accuracy and repeatability of inspection results.
- The issue of sound velocity variation and mode conversion: The speed of sound in coatings typically differs from that in steel, and non-uniform coating thickness introduces measurement errors. At the coating-to-steel interface, ultrasonic waves may undergo complex mode conversion—such as longitudinal waves transforming into transverse waves—rendering signal analysis more intricate.
- Near-surface blind zone issue: Due to the width of the transmitted pulse and the influence of interface echoes, conventional UT exhibits a certain degree of detection blindness in near-surface regions. The presence of coatings may exacerbate this blind zone problem.

#### 3.5.2. Recent Advances

In recent years, with the advancement of ultrasonic technology, significant progress has been made in the flexibility and imaging capabilities of UT. To meet the demands of practical production processes, numerous scholars have dedicated themselves to applying this technology to the detection of metal defects beneath coatings. Santo et al. [86] applied ultrasonic testing to detect weld defects in in-service ships. Through experiments, they compared defect detection capabilities on welds without coatings versus those covered by a 740 μm-thick paint layer. Radiographic testing was used to validate the ultrasonic results. The results demonstrated that ultrasonic testing could detect a 50 mm-long crack located 5.6 mm beneath the metal surface without coating removal, meeting the requirements for ship weld defect detection. Predoi et al. [87] applied UT to inspect pipeline welds with 50 μm coatings, investigating paint coatings’ impact on pipeline weld ultrasonic inspection. Experiments demonstrated that longitudinal wave attenuation increases with frequency. Shirahata et al. [88] employed UT to monitor fatigue crack initiation in flange gusset plate welds without coating removal. The results confirmed ultrasonic detection of 1.1 mm in width and 1.6 mm in length fatigue cracks beneath 280–430 μm coatings.

Some scholars have also conducted extensive research in signal processing. Chen et al. [49] proposed a full-matrix ultrasonic imaging method for detecting pipeline weld defects beneath protective coatings, employing coded excitation and multimode wavenumber domain image reconstruction techniques. This technique combines nonlinear frequency modulation (NLFM) with Golay codes to form encoded ultrasonic excitation sequences, achieving superior penetration and side lobe suppression. It effectively addresses the complex wave propagation and image migration challenges posed by welds covered by protective coatings. Experimental results demonstrate the method’s capability to detect pipeline weld defects beneath 2.6 mm coatings, including incomplete fusion, incomplete penetration, slag inclusions, and cracks, with a minimum detectable defect size of 2mm in width and 4mm in length. Wu et al. [89] proposed an extended phase shift migration (EPSM)-based full-matrix imaging scheme for coating-covered steel defect detection, overcoming interlayer acoustic velocity variations and interface refraction phenomena in dual-layer structures. In this approach, based on the phase shift migration theory for single static pulse-echo imaging, an EPSM-based full matrix imaging method is derived through extrapolation of the transmitted and received wavefields. This enables the acquisition of focused images of multilayer structures with higher lateral resolution. Experiments demonstrate that this method can detect Ø1 mm steel porosity defects beneath a 20 mm coating, with detection capability and accuracy significantly surpassing those of the Total Focusing Method (TFM). Dumrongkit et al. [90] proposed equations and transmission correction factors to compensate for coating-induced energy attenuation during ultrasonic testing of coated steel welds. Experiments validated the effects of coating thickness (0–900 μm), probe angle, and frequency on crack and lack-of-fusion defect detection accuracy. The results indicated that energy attenuation in angle probes increased with both angle and frequency. When coating thickness exceeded half the acoustic wavelength, further increases led to greater energy attenuation and reflection.

In conclusion, UT stands as one of the preferred methods for detecting internal defects in welds. It is extensively employed in the non-destructive examination of critical welds within boilers, pressure vessels, and pipelines. This technique demonstrates high detection accuracy and strong quantitative capabilities when inspecting welds beneath coatings. However, it typically requires coupling agents, specific surface preparation, and signal processing operations.

### 3.6. Electromagnetic Acoustic Transducer (EMAT)

#### 3.6.1. Inspection Principle

**Figure 6:**
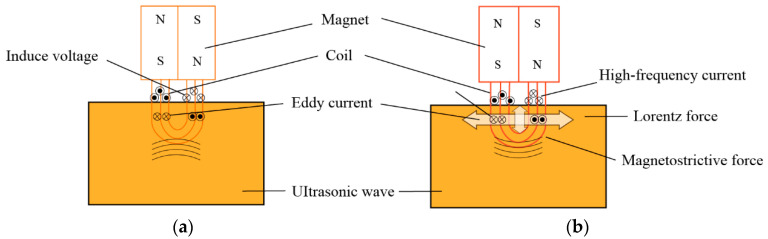
Principle of EMAT [96]. (**a**) Transmitted wave; (**b**) Reflected wave.

EMAT is a non-contact ultrasonic testing technique. It employs a static magnetic field and dynamic alternating current applied near the surface of conductive materials, utilizing the Lorentz force (for non-ferromagnetic materials) or the magnetostrictive effect (for ferromagnetic materials) to directly excite and receive ultrasonic waves within the material [91], as illustrated in Figure 6. EMAT technology requires no coupling agent, enabling direct inspection on non-conductive coatings. This eliminates coupling-related issues and, theoretically, the generated ultrasonic waves are excited within the steel substrate, meaning that the non-conductive coating itself does not constitute a penetration barrier [92]. However, in practical engineering applications, its relatively low energy conversion efficiency results in weaker ultrasonic signals, demanding higher signal-to-noise ratio specifications from the inspection system [93,94]. EMATs can excite multiple ultrasonic modes, including transverse waves, longitudinal waves, Lamb waves, and SH waves, enabling detection of surface and internal defects such as cracks, lack of fusion, and slag inclusions. Detection accuracy depends on ultrasonic frequency, wavelength, sensor design, and defect type, typically achieving millimeter-level precision [95]. Furthermore, EMATs exhibit a certain tolerance to surface roughness, provided the tested surface is relatively clean and free from large loose deposits.

#### 3.6.2. Recent Advances

Early EMAT technology faced developmental constraints due to low conversion efficiency and weak signals. However, in recent years, high-performance magnets and novel coil designs—such as helical coils and interdigitated coils—have significantly enhanced EMAT sensitivity. Concurrently, advances in electronic technology have enabled high-frequency, high-power excitation, further amplifying EMAT signals. Presently, the pronounced lift-off effect remains the foremost challenge requiring resolution in EMAT applications, with numerous scholars contributing to its investigation.

Deng et al. [50] proposed an imaging algorithm based on Synthetic Aperture Focusing Technology (SAFT) to detect subsurface defects in coated metallic structures. This algorithm processes surface wave signals from array-picked-up electromagnetic acoustic transducers (EMATs) to enhance signal-to-noise ratio and subsurface defect detection capability, validated through simulations and experiments. The algorithm calculates the focal echo arrival times received by each pickup sensor, filters corresponding echo amplitudes, and performs time-based stacking to generate amplified echo signals. By treating each grid node as a virtual focal point, amplified echo amplitudes are computed individually. The amplified signal intensities are then visualized as color-coded contour lines around grid nodes, enabling defect enhancement imaging. The results demonstrate the method’s ability to effectively detect a crack measuring 0.2 mm wide, 4 mm long, and 4 mm deep at a depth of 0.5 mm below the surface. He et al. [51] proposed a method to generate fundamentally symmetric Lamb waves on ferromagnetic structures and designed a pure-coil EMAT to address the issue of magnetic attraction affecting measurements. This sensor employs a “transmit-receive” configuration: the transmit coil consists of 10 turns of Ø0.68 mm enameled wire wound on a Ø38 × 20 mm plastic model, while the receive coil comprises 40 turns of Ø0.1 mm enameled wire wound on a Ø38 × 20 mm permanent magnet. The experimental results indicate that the magnetic force generated by the EMAT and detection accuracy reach equilibrium at a lift-off distance of 19 mm. Wu et al. [52] employed a five-factor, four-level orthogonal experimental design based on the peak ultrasonic signal amplitude and lift-off decay curve to investigate the influence of structural parameters on EMAT performance. This approach aimed to suppress lift-off effects caused by surface irregularities and uneven coating thickness on the inspected surface. The study utilized the orthogonal experimental method to examine the impact of five structural parameters on EMAT performance. Results indicate that nearly all factors significantly affect the theoretical maximum amplitude, while only the distance between the magnet and coil effectively influences lift-off performance. Experimentally, when lift-off reached 1 mm, conversion efficiency increased by 125%. Additionally, lift-off performance and theoretical maximum amplitude improved by 44.9% and 64.9%, respectively, effectively suppressing the lift-off effect. Si et al. [53] proposed a Variational Modal Decomposition combined with Wavelet Denoising (VMD) method to address the issues of low conversion efficiency and susceptibility to noise in EMATs. This algorithm transforms the modal decomposition problem into a variational optimization problem and employs the Alternating Direction Method of Multipliers (ADMM) for parameter optimization. During optimization, a set of band-limited intrinsic mode functions (IMFs) is obtained. Experiments demonstrate that this method can simultaneously suppress both high-frequency narrowband noise and normal noise in EMAT signals under lift-off detection conditions. At a lift-off height of 1.3 mm, the detection error for a defect measuring 20 mm in length and 2 mm in width and depth is 1.6%.

In general, the application of EMAT in industry is gradually expanding. As the technology matures, EMAT holds promise as a significant method for detecting metal defects beneath coatings. It should be noted that EMAT imposes certain requirements on the surface finish and material homogeneity of workpieces, as deviations may adversely affect detection accuracy.

### 3.7. Microwave Testing (MWT)

#### 3.7.1. Inspection Principle

**Figure 7:**
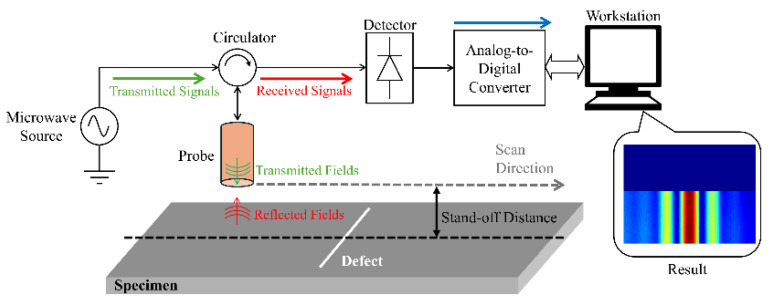
MWT Operating Principle [102].

Microwave Testing is a non-contact electromagnetic inspection technique operating within the frequency range of 300 MHz to 300 GHz. It exhibits excellent penetration capabilities for non-metallic coatings and composite materials, whilst remaining unaffected by high attenuation [97,98,99]. Upon traversing dielectric coatings, a portion of the microwave energy reflects at the coating–metal interface, whilst another fraction may penetrate the coating and reflect back from internal defects within the metal substrate. By emitting microwaves and analyzing the reflected, transmitted, or scattered signals, the internal structure and defect conditions of materials can be inferred, as illustrated in Figure 7. Microwave detection is highly sensitive to material discontinuities. Moreover, microwaves struggle to penetrate the interior of good conductors beyond very shallow surface layers. Consequently, this method is exceptionally well-suited for detecting surface defects in steel structures [100,101].

#### 3.7.2. Recent Advances

For coated metallic structures, microwave inspection can be employed to detect defects within the coating itself, as well as defects or thickness variations in the underlying metal substrate beneath the coating. This paper focuses specifically on the latter. To mitigate the influence of crack orientation on detection results, Abou-Khousa et al. [103] designed a single/dual-polarized circular aperture antenna combined with synthetic aperture imaging technology. This approach detects cracks oriented in various directions beneath 4.25 mm-thick rubber and 12.7 mm-thick PTFE coatings. The designed antenna enables both single-static synthetic aperture radar imaging through co-polarized wave reflection measurements between ports and cross-polarized imaging via transmission measurements between ports. Experiments demonstrate that at 24 GHz, this method significantly suppresses background clutter and dielectric layer effects, ensuring high detection accuracy for crack size and orientation.

In order to suppress interference caused by coatings, Rahman et al. [42] employed dual-polarized wave detection to mitigate background interference. This approach leverages the principle that cross-polarized measurements remain unaffected by surface irregularities, enabling crack detection beneath inclined surfaces and thick, irregular coatings. Experiments were conducted on metal blocks coated with 6.25 mm thick polytetrafluoroethylene (PTFE) and 6 mm thick SOFI material. Results demonstrated that at 25 GHz, this method effectively suppressed coating interference and successfully detected a crack measuring 0.25 mm in width and 40 mm in length. Gao et al. [104] designed a W-band (75–110 GHz) differential probe system incorporating a fast-response broadband signal source operating in the 13–18 GHz frequency range. The output signal underwent frequency doubling and tripling to achieve an output range of approximately 78–108 GHz, enabling crack detection near aircraft fuselage fasteners and beneath paint coatings. Studies demonstrated the system’s effective suppression of coating effects, successfully detecting 2 mm surface cracks on overlapping metal plates beneath 0.1 mm paint layers. Ur Rahman et al. [105] investigated the crack detection capability of a UHF probe under coatings of varying materials and thicknesses. This probe consists of an 8-turn spiral resonator (SR) fabricated on an FR4 substrate, with its specific structure detailed in [106]. The results demonstrated that at 783 MHz, the probe successfully detected a metal crack measuring 0.25 mm in width and 10 mm in length beneath a 3 mm thick rubber coating, fully validating its superior performance.

In order to enhance detection sensitivity and resolution, Haryono et al. [106] proposed a planar resonator probe to achieve higher resolution at lower frequencies in near-field microwave inspection. This probe consists of an eight-turn square SR antenna fabricated on a printed circuit board (PCB) using an FR4 substrate. Measuring 6 mm in width and 6 mm in length, the probe employs a matching network comprising an adjustable capacitor (3–8 pF) and two equivalent ceramic capacitors (2 pF) to achieve 50-ohm impedance matching. It delivers imaging resolution within the millimeter range, exhibits polarization-independent response, and maintains high overall sensitivity. The probe’s performance was comprehensively evaluated through numerical simulation and experimental measurements. Results demonstrate that it achieves 3 mm resolution imaging at an operating frequency of 783 MHz. Furthermore, the probe was successfully applied to detect metal corrosion and cracks beneath coatings, clearly identifying a Ø3 × 0.5 mm defect under 1 mm of paint coverage. Rahman et al. [107] proposed a resonant iris circular aperture probe operating at 26 GHz to further improve near-field imaging resolution and sensitivity. This probe is designed based on a waveguide-fed circular aperture probe, incorporating an additional resonant iris structure at its aperture, resulting in a simple and practical design. Research indicates that compared to an unloaded circular aperture probe, this probe exhibits higher resolution and sensitivity. Specifically, for crack detection, the proposed probe achieves over twofold and threefold improvements in resolution and sensitivity, respectively. For corrosion detection beneath 1 mm paint coatings, it delivers a 2–5 times higher image signal-to-noise ratio than the bare circular aperture probe, enabling detection of 0.25 mm-wide metal cracks concealed under 1 mm paint layers.

The aforementioned research indicates that MWT technology holds significant potential for detecting surface cracks, cracks beneath coatings, and surface cracks in corroded conditions. Moreover, the operational frequency for detecting metal defects beneath millimeter-thick coatings is concentrated below 30 GHz. This limitation arises because millimeter waves (30–300 GHz) suffer significant signal loss when inspecting metal defects beneath coatings, rendering them unsuitable for such applications. Consequently, while microwave detection excels in non-contact, high-speed imaging, it remains nearly incapable of detecting minute internal cracks in metals. With future advancements in high-resolution microwave imaging technology, MWT is anticipated to play an increasingly significant role in the remote monitoring and large-scale screening of defects beneath coatings.

### 3.8. Eddy Current Pulse Thermography (ECPT)

#### 3.8.1. Inspection Principle

**Figure 8:**
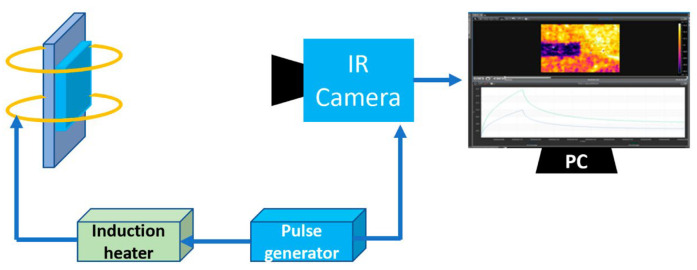
Principle of ECPT [115].

Eddy Current Pulse Thermography is an enhanced form of eddy current thermal imaging characterized by the use of short, high-energy pulsed currents to excite coils, rather than continuous sinusoidal currents [108,109]. This pulsed excitation instantly induces powerful eddy currents within the workpiece, causing its surface to heat rapidly; excitation is then ceased to observe the subsequent cooling process. Should defects be present, the thermal diffusion behavior within the defective area differs from the surrounding material, resulting in anomalous cooling curves recorded by the infrared thermal imager [110,111,112,113,114]. By analyzing the transient thermal image sequence following pulse heating, information regarding defect depth and size can be obtained, as illustrated in Figure 8. ECPT combines the deep penetration capability of pulsed eddy currents with the intuitive imaging advantages of thermal imaging. This technique is equally applicable to inspecting coated structures. Utilizing pulsed excitation allows the low-frequency component to penetrate thicker coatings and reach greater depths within the metal, thereby detecting defects at deeper levels.

#### 3.8.2. Recent Advances

During the detection of surface defects in industrial welds, complex surface conditions readily induce eddy current field variations and temperature anomalies that interfere with detected defect signals. The presence of coatings further complicates detection, as thermal signals become extremely faint during penetration through the coating due to diffusion and attenuation, rendering defect detection challenging. Scholars have conducted extensive research to address this challenge. Tang et al. [43] addressed interference caused by weld surface irregularities by proposing a symmetrical induction excitation probe based on the eddy current pulse thermography (ECPT) method. This probe is suitable for detecting weld defects beneath coatings and was applied to train bogie weld defect detection. They also constructed a simulation model for detecting weld defects under coatings. This probe utilizes a yoke coil to generate a uniform excitation magnetic field within the excitation zone, enabling uniform heating of the inspected surface without obstructing the field of view. Experiments validated the detection effectiveness of weld cracks and porosity defects under varying coating thicknesses (100–300 μm) while confirming its capability to detect different crack geometries. The results demonstrate that the probe successfully detects 0.15 mm-wide, 1 mm-deep, 3–5 mm-long cracks oriented in various directions and Ø3 × 1 mm porosity defects beneath 0.3 mm paint coverage at a scanning speed of 20 mm/s.

Some scholars have applied the finite element method to simulate and validate new approaches based on ECPT technology. Chen et al. [116] employed pulsed ECPT to detect cracks beneath steel structure coatings. In this study, numerical simulations analyzed the electromagnetic and thermal responses of steel structures with covered cracks during induction heating, extracting thermal imaging signatures generated by covered cracks. Results demonstrated that these thermal signatures effectively detected cracks measuring 0.5 mm in width and depth, and 10 mm in length within steel structures coated with 0.5 mm epoxy resin. In another study by this scholar [117], pulsed eddy current thermal imaging was employed to simultaneously detect adjacent and overlapping cracks/interface defects in carbon fiber-reinforced polymer/steel composite (ABCSS) materials, with both simulation and experimental validation conducted. Numerical simulations revealed that cracks in the steel matrix simultaneously affect eddy current distribution and heat transfer. However, interfacial defects (delamination or debonding) do not influence eddy current distribution but do affect heat transfer. Experimental results validated the simulation accuracy, demonstrating the technique’s ability to effectively detect adjacent and overlapping cracks/debonding and crack/delamination defects in ABCSS materials. The tested crack dimensions were 2 mm wide, 2 mm deep, and 30 mm long, while the delamination defect was 10 mm wide, 0.2 mm deep, and 30 mm long. This validation confirms the effectiveness of pulsed eddy current thermal imaging for simultaneously detecting both matrix and interface defects.

Additionally, Wang et al. [118] investigated the influence of polymer coatings on ECPT of surface cracks in steel structures. First, thermal characteristics of typical surface cracks were analyzed via numerical simulation. Subsequently, experiments examined the impact of coatings of varying thicknesses on artificial crack detection. Finally, coating effects were analyzed using specimens with minute natural cracks. Results indicated that artificial cracks measuring 5 mm in length, 3 mm in depth, and 0.5 mm in width remained detectable beneath 530 μm thick coatings. However, for testing specimens with minute natural cracks nearly invisible to the naked eye, the maximum effective coating thickness for this method is only 175 μm.

Some researchers have also applied artificial intelligence technologies to ECPT. Zhang et al. [119] proposed a method combining the YOLOv5 target detection model with ECPT technology to accurately detect defects in coated steel structures. Validation demonstrated that this approach can accurately identify and classify matrix cracks, delamination, and corrosion defects within coated steel structures, achieving detection accuracy exceeding 95%.

From the above research, it is evident that, on the one hand, the current application of ECPT technology for detecting weld defects beneath coatings is largely confined to surface or near-surface defects, with further research required for the detection of internal defects. On the other hand, methods combining artificial intelligence with inspection techniques demonstrate considerable potential. Furthermore, owing to the requirement for high-power pulsed power supplies and high-speed infrared cameras, ECPT equipment is presently chiefly employed in research and high-end inspection domains. Looking ahead, as equipment becomes more compact and costs decrease, its adoption is anticipated to expand into industrial settings, emerging as an effective method for detecting weld defects in coated steel structures.

## 4. Discussion

To achieve weld defect detection in steel structures while preserving coatings, this paper details the research progress of NDT techniques capable of detecting defects in coated steel structure welds. It focuses on summarizing and organizing the detection principles, detection capabilities, coating penetration capabilities, primary advantages, and limitations of different methods. The detection capabilities and coating penetration capabilities of various methods are presented in Table 8, while their advantages and limitations are summarized in Table 9. Compared to traditional NDT techniques, most of these methods are based on technologies such as electromagnetic induction, electromagnetic wave propagation, acoustics, and infrared thermal imaging. They enable defect detection without compromising environmental safety or the personal safety of construction personnel, while offering higher detection efficiency and lower inspection costs.

From the above comparison, it is evident that methods possessing rapid detection capabilities for surface and near-surface defects in coated steel structural welds, coupled with favorable localization and quantification abilities, include ECT, PEC, MFL, ACFM, MWT, and ECPT. Among these, both ECT and PEC technologies are based on electromagnetic induction principles, detecting defects by measuring changes in impedance or induced voltage in the coil caused by eddy current variations. The primary difference between the two lies in their excitation methods. Consequently, while both technologies can detect metallic defects beneath coatings, ECT’s detection accuracy and sensitivity are susceptible to variations in coating thickness and inspection frequency. In contrast, PEC is less constrained by these factors, can detect deeper defects, and offers capabilities for large-area, rapid inspection. However, PEC exhibits slightly lower sensitivity for planar defects compared to volumetric defects. Consequently, it is more suitable for detecting volumetric defects in coated steel structural welds, such as porosity and slag inclusions.

Based on their detection principles, both MFL and ACFM technologies operate on the principle of electromagnetic induction, determining defect location and size by detecting changes in leakage magnetic fields. The primary differences between the two lies in their excitation sources and measurement parameters. MFL employs a direct-current electromagnetic field as its excitation source, primarily determining defect location and estimating defect size by measuring the intensity of the leakage magnetic field. ACFM utilizes low-frequency alternating current as its excitation source, determining defect location and size by detecting changes in the phase and amplitude of the magnetic field components. Consequently, MFL is only suitable for detecting surface or near-surface defects in ferromagnetic materials. It requires magnetizing the material being tested, and the results are influenced by the material’s magnetic permeability. ACFM, however, is suitable for detecting surface defects in all conductive metallic materials and offers higher quantitative accuracy. It can determine not only defect length but also defect depth, making it more suitable for detecting area-type defects on coated steel structures, such as cracks, lack of fusion, and dense porosity.

Additionally, while MWT and ECPT technologies both offer advantages such as non-contact operation, imaging capability, and rapid detection speed, they are often constrained by spatial limitations during defect detection in complex structures due to their relatively complex equipment. These methods are better suited for rapid scanning of external, open areas of steel structures and are not ideal for detecting defects in internal, confined spaces. Consequently, both approaches face challenges in practical applications for detecting weld defects in coated steel structures.

According to the survey findings, the only methods capable of detecting internal defects within steel structures are ultrasonic-based inspection technologies, including UT and EMAT. Both possess precise detection capabilities for internal defects in metallic materials, with their differences also reflected in their excitation methods. UT technology transmits ultrasonic waves generated by the inspection probe into the test specimen via contact, thus requiring coupling, which imposes high demands on the surface being inspected. EMAT, however, generates ultrasonic waves directly within the metal substrate through non-contact excitation. This approach effectively mitigates the influence of non-conductive coatings, avoids coupling-related issues, and offers rapid detection capabilities. Consequently, EMAT is more suitable for the swift detection of internal defects in welded joints of coated steel structures.

The above analysis demonstrates that different NDT techniques possess distinct advantages and applicable scopes. Therefore, in practical applications, the selection of appropriate methods must be based on factors such as coating characteristics, defect types, inspection accuracy requirements, and the structure of the inspected product. Sometimes, multiple methods need to be combined to enhance inspection reliability. For instance, ACFM technology can be selected for detecting surface-type defects on welded surfaces of coated steel structures. PEC technology is suitable for detecting volume-type defects on the weld surface. EMAT technology can be chosen for detecting internal defects within the weld.

## 5. Future Development Trends

For the future, NDT techniques for welds beneath coatings will evolve towards greater efficiency, intelligence and automation. The following aspects warrant attention:

1. More Advanced NDT Technologies: With technological advancements, novel NDT principles and techniques will be explored and applied to coating-under inspection. For instance, ACFM technology utilizing pulse excitation can enhance detection capabilities for subsurface defects while promising improved quantitative assessment of volumetric defects; Acoustic Emission (AE) testing can monitor the propagation of active defects such as cracks in real-time during structural service, with future potential for integration with wireless sensing technology to enable long-term online monitoring of coated welds; Arrayed Eddy Current Testing (AECT) technology covers larger inspection areas and generates two-dimensional weld images, facilitating more intuitive defect location identification beneath coatings; Phased Array Ultrasonic Testing (PAUT) can effectively detect internal structural defects while preserving coatings through the use of specialized delay blocks or wedges and appropriate parameter optimization. The advancement of these emerging technologies will further diversify the means of coating-under inspection and enhance the capability to identify complex defects.
2. Artificial Intelligence and Big Data: Artificial intelligence and big data analytics will play an increasingly vital role in the field of NDT. On the one hand, machine learning algorithms can be employed to automatically analyze NDT signals and identify defects, thereby reducing human misjudgments. For instance, deep learning-based image recognition technology can be utilized to automatically detect defects in microwave imaging or ultrasonic images. Concurrently, the accumulation and analysis of big data enable the establishment of defect characteristic databases and life prediction models, facilitating intelligent diagnostics and trend forecasting for weld defects beneath coatings. For instance, integrating multiple inspection datasets with structural stress models can predict crack propagation rates and remaining service life, providing scientific grounds for maintenance decisions. Artificial intelligence may also be applied to optimize inspection processes, such as automatically adjusting probe parameters and path planning, thereby enhancing both efficiency and accuracy.
3. Robotics and Automated Inspection: Advances in robotic technology have provided new platforms for NDT. Various crawling robots, unmanned aerial vehicles (UAVs), and similar devices can carry NDT probes into areas that are difficult or hazardous for humans to access. For instance, crawling robots can autonomously traverse the exteriors of coated steel structures inaccessible to personnel, performing defect detection tasks; UAVs equipped with electromagnetic or optical sensors can conduct inspections of elevated steel structures. These automated systems enable inspections without halting operations or erecting scaffolding, enhancing both safety and efficiency. Future inspection robots will become more intelligent, featuring environmental awareness, autonomous navigation, and adaptive inspection capabilities. They will automatically adjust inspection strategies based on structural geometry and coating conditions. Furthermore, robotic inspection facilitates real-time data transmission and remote monitoring, allowing specialists to provide on-site guidance remotely and improve diagnostic accuracy.
4. Multi-sensor fusion and integrated inspection: Single inspection methods often have inherent limitations. The future trend lies in employing multi-sensor information fusion technology to comprehensively analyze data obtained from different inspection methods, thereby enabling a more thorough and accurate assessment of weld quality. For instance, integrating ultrasonic and eddy current inspection results allows simultaneous detection of both internal and surface defects. Multi-sensor systems can also cross-validate each other, enhancing inspection reliability. In practical applications, integrated inspection equipment incorporating multiple NDT functions can be developed according to specific requirements, enabling multi-faceted assessment of welds beneath coatings in a single scan. Such integrated inspection systems represent a key future development direction for NDT.
5. Standards and Training: As coating-under-inspection technologies advance and gain wider application, corresponding standards must be refined accordingly. Future efforts should focus on developing more comprehensive standards for NDT of coated steel structures, clearly defining the scope of application, acceptance criteria, and quality control requirements for various methods to provide a basis for engineering implementation. Concurrently, greater emphasis must be placed on training inspection personnel, cultivating multi-skilled professionals proficient in diverse new technologies to align with the trend towards intelligent and automated NDT.

In conclusion, the future of NDT for welded joints in coated steel structures will continue to advance under the impetus of new technologies and methodologies, progressing towards higher sensitivity, greater precision, and increased automation. The integration of artificial intelligence and robotics will enable NDT to evolve from traditional manual qualitative inspections towards intelligent quantitative monitoring, thereby providing more robust safeguards for the safe operation of steel structures.

## 6. Conclusions

Steel structures, as vital components of modern infrastructure, demand paramount attention to safety and durability. While protective coatings effectively shield these structures from environmental corrosion, they also present significant technical challenges for the NDT of weld defects. This paper systematically reviews the fundamental principles and research advancements in NDT technologies, specifically targeting the non-destructive testing of weld defects in coated steel structures.

1. Common types of weld defects in steel structures, their causes, and preventive measures are elaborated. Challenges in NDT for coated steel welds and potential countermeasures are analyzed.
2. Eight key technologies—ECT, PEC, MFL, ACFM, UT, EMAT, MWT, and ECPT—are examined under coated conditions. Their fundamental principles, application advantages, limitations, recent breakthroughs, and future prospects are evaluated.
3. Research findings indicate that electromagnetic methods (eddy current, pulsed eddy current, magnetic flux leakage, etc.) can detect surface and near-surface defects without coating damage. Ultrasonic methods (conventional ultrasonics, EMAT, etc.) excel at detecting internal defects but are sensitive to coupling and coatings. Optical and thermal imaging methods (pulse eddy current thermal imaging, etc.) offer novel approaches and tools for detecting metal defects beneath coatings. However, a single technique often cannot perfectly address all issues. A “customized, multi-technology integration” strategy tailored to specific coating characteristics, defect types, and inspection requirements represents the future development direction.
4. For weld defect detection in coated steel structures within aerospace applications, ACFM technology demonstrates high detection efficiency and precise localization/quantification for surface and near-surface defects. For internal metallic defects, EMAT technology achieves high detection efficiency and accuracy without requiring coupling.

With the emergence of new materials and processes alongside advancements in detection technologies, NDT for welds beneath coatings will continue to evolve. The emergence of novel inspection technologies, deep integration of artificial intelligence, and application of robotic automation will significantly enhance inspection efficiency and accuracy.

Future research on coating-covered steel structure weld defect detection should focus on:

- Developing novel sensors and transducers;
- Advancing multi-modal information fusion techniques;
- Continuously innovating AI algorithms (e.g., few-shot learning, explainable AI) and the integrated application of cutting-edge technologies like digital twins.

This will drive the transformation of NDT from passive inspection to proactive monitoring, and from manual interpretation to intelligent diagnosis. It will enhance the accuracy of locating and quantitatively detecting various defect types, thereby safeguarding the safety of steel structures throughout their entire lifecycle. It is hoped that personnel involved in coating inspection and technicians unfamiliar with NDT capabilities for detecting metal defects beneath coatings will gain a better understanding through this research. This will facilitate the selection of appropriate inspection techniques, thereby improving the accuracy and efficiency of metal defect detection beneath coatings. Ultimately, these efforts will promote the widespread application of advanced NDT technologies in the field of metal defect detection beneath coatings, holding profound significance for maintaining the safety of facilities and equipment and extending their service life.

## Figures and Tables

**Table 1:** Geometric parameters of excitation coils and detection coils [39].

Item	Cylindrical Probe		U-Shaped Probe	
	Excitation Coil	Receiving Coil	Excitation Coil	Receiving Coil
Wire diameter/mm	0.6	0.3	0.6	0.3
Number of turns	80	200	80	200
Inner diameter/mm	20	8	5	8
External diameter/mm	40	15	8	14
Height/mm	23	6.5	13	6.5

**Table 2:** Probe parameters [46].

Probe No.	Coil Type	Wire Diameter (mm)	Number of Turns	Inner Diameter (mm)	Outer Diameter (mm)	Coil Height (mm)	Coil Pitch (mm)
P1	excitation coil	0.6	300	15	30	15	30
	detection coil	0.35	900	15	30	15	
P2	excitation coil	0.8	700	30	60	30	60
	detection coil	0.4	2800	30	60	30	

**Table 3:** Dimensions of Three Artificial Rectangular Groove Defects [41].

Defect No.	Length (mm)	Width (mm)	Depth (mm)
1#	25.4	25.4	6.35
2#	50.8	12.7	5.08
3#	38.1	38.1	5.08

**Table 4:** Size of the defects [70].

Defect No.	Length (mm)	Width (mm)	Depth (mm)	Defect No.	Length (mm)	Width (mm)	Depth (mm)
1#	30	1	6	7#	30	4	1
2#	30	2	6	8#	30	4	2
3#	30	3	6	9#	30	4	3
4#	30	4	6	10#	30	4	4
5#	30	5	6	11#	30	4	5
6#	30	6	6	12#	30	4	6

**Table 5:** Crack dimensions [48].

Crack No.	Length (mm)	Width (mm)	Depth (mm)	Crack No.	Length (mm)	Width (mm)	Depth (mm)
1#	30	2	2	6#	25	2	3
2#	30	2	5	7#	20	2	3
3#	30	2	6	8#	15	2	3
4#	30	2	7	9#	10	2	3
5#	30	2	8	10#	5	2	3

**Table 6:** Crack dimensions [78].

Crack No.	Length (mm)	Width (mm)	Depth (mm)	Crack No.	Length (mm)	Width (mm)	Depth (mm)
1#	12	0.5	1	5#	12	0.5	3
2#	12	0.5	3	6#	16	0.5	3
3#	12	0.5	5	7#	20	0.5	3
4#	12	0.5	7	8#	24	0.5	3

**Table 7:** Crack dimensions [79].

Crack No.	Length (mm)	Width (mm)	Depth (mm)	Crack No.	Length (mm)	Width (mm)	Depth (mm)
1#	30	0.8	5	10#	20	0.8	1
2#	30	0.8	4	11#	10	0.8	5
3#	30	0.8	3	12#	10	0.8	4
4#	30	0.8	2	13#	10	0.8	3
5#	30	0.8	1	14#	10	0.8	2
6#	20	0.8	5	15#	10	0.8	5
7#	20	0.8	4	16#	15	0.8	5
8#	20	0.8	3	17#	5	0.8	5
9#	20	0.8	2	18#	25	0.8	5

**Table 8:** Detection capabilities and coating penetration of different NDT techniques.

NDT Technique	Detection Capability	Maximum Penetration Coating Thickness
ECT	Detectable small defects on surfaces and near-surface areas (<1 mm)	10 mm non-conductive coating
PEC	Capable of assessing metal loss and defects to a certain depth	14 mm non-conductive coating (base material defects detectable beneath 35 mm non-metallic layers via specially designed sensors)
MFL	Detectable surface/near-surface defects	5 mm non-conductive coating
ACFM	Capable of detecting surface cracks with quantitative assessment of crack length and depth	5.5 mm non-conductive coating
UT	Capable of detecting internal defects with precise localization and quantification	With the improved method, defects in the substrate beneath a 20 mm non-conductive layer can be detected.
EMAT	Comparable to UT	19 mm non-conductive coating
MWT	Detectable surface cracks, metal loss, etc.	17 mm Non-metallic composite layer
ECPT	Detectable subsurface defects	0.5 mm Non-metallic composite layer

**Table 9:** Advantages and limitations of different NDT techniques.

NDT Technique	Advantages	Limitations	Future Perspective
ECT	Non-contact Capable of measuring coating thickness	Affected by lift-off Insensitive to deep defects Temperature drift needs to be considered	Multi-frequency signal fusion; Enhanced quantitative identification capability; Probe miniaturization and array design; Development of lift-off effect compensation technology.
PEC	Non-contact Fast detection Able to detect deep defects Insensitive to coating thickness variations	Low sensitivity to surface defects	Enhance defect detection sensitivity and accuracy; Develop defect visualization technology; Design broadband excitation sources.
MFL	Simple equipment Fast detection speed Suitable for large area defect detection	Requires magnetization of the metal substrate to be measured Insensitive to buried defects	Optimization of magnetic field uniformity; Enhancement of signal-to-noise ratio; Development of compact, high-resolution probes;
ACFM	Simple equipment Rapid testing speed Intuitive results	Applicable only to surface cracks Ineffective against internal defects	Reduce sensitivity to lift-off variations; Enhance detection capability for irregular welds.
UT	Sensitive to internal defects Quantifiable Suitable for thick workpieces	Coupling is required and sensitive to surface coatings	Enhance detection efficiency; Reduce interface effects; Improve coupling conditions.
EMAT	Non-contact No coupling required Faster detection speed than UT	Signal is weak and requires special handling	Improve signal-to-noise ratio; Enhance transducer efficiency; Increase defect detection depth.
MWT	Non-contact Imaging capability Sensitive to surface defects	Insensitive to small cracks within the metal Complex equipment	Reduce sensitivity to variations in lift-off distance; Improve penetration capability to expand detection range; Develop portable devices.
ECPT	Detects deep-seated defects Rapid imaging	Complex equipment Requires high power supply Difficult to quantify	Enhance detection efficiency; Improve quantitative defect detection capabilities; Design low-power, high-power-density excitation sources.

## Data Availability

No new data were created or analyzed in this study. Data sharing is not applicable to this article.
